# Onion (*Allium cepa* L.) Skin: A Rich Resource of Biomolecules for the Sustainable Production of Colored Biofunctional Textiles

**DOI:** 10.3390/molecules24030634

**Published:** 2019-02-11

**Authors:** Lucia Pucciarini, Federica Ianni, Valentina Petesse, Federica Pellati, Virginia Brighenti, Claudia Volpi, Marco Gargaro, Benedetto Natalini, Catia Clementi, Roccaldo Sardella

**Affiliations:** 1 Department of Pharmaceutical Sciences, University of Perugia, Via Fabretti 48, 06123 Perugia, Italy; lucia.pucciarini@hotmail.it (L.P.); federica.ianni@chimfarm.unipg.it (F.I.); benedetto.natalini@unipg.it (B.N.); roccaldo.sardella@unipg.it (R.S.); 2 Department of Chemistry Biology and Biotechnology, University of Perugia, Via Elce di Sotto 8, 06123 Perugia, Italy; valentinapetesse@gmail.com; 3 Department of Life Sciences, University of Modena and Reggio Emilia, Via G. Campi 103, 41125 Modena, Italy; federica.pellati@unimore.it (F.P.); virginia.brighenti@unimore.it (V.B.); 4 Department of Experimental Medicine, University of Perugia, P.le Severi, 06132 Perugia, Italy; claudia.volpi@unipg.it (C.V.); marco.gargaro@unipg.it (M.G.)

**Keywords:** *Allium cepa* L., onion skin, phenolics, antioxidant activity, biofunctional textiles, photo-oxidative damage

## Abstract

The aqueous extract of dry onion skin waste from the ‘Dorata di Parma’ cultivar was tested as a new source of biomolecules for the production of colored and biofunctional wool yarns, through environmentally friendly dyeing procedures. Specific attention was paid to the antioxidant and UV protection properties of the resulting textiles. On the basis of spectrophotometric and mass spectrometry analyses, the obtained deep red-brown color was assigned to quercetin and its glycoside derivatives. The Folin–Ciocalteu method revealed good phenol uptakes on the wool fiber (higher than 27% for the textile after the first dyeing cycle), with respect to the original total content estimated in the water extract (78.50 ± 2.49 mg equivalent gallic acid/g onion skin). The manufactured materials showed remarkable antioxidant activity and ability to protect human skin against lipid peroxidation following UV radiation: 7.65 ± 1.43 (FRAP assay) and 13.60 (ORAC assay) mg equivalent trolox/g textile; lipid peroxidation inhibition up to 89.37%. This photoprotective and antioxidant activity were therefore ascribed to the polyphenol pool contained in the outer dried gold skins of onion. It is worth noting that citofluorimetric analysis demonstrated that the aqueous extract does not have a significative influence on cell viability, neither is capable of inducing a proapoptotic effect.

## 1. Introduction

Biofunctional textiles, also known as ‘biomedical textiles’, and most frequently as ‘cosmetotextiles’ are opportunely engineered materials able to exert one or more beneficial healthy effects on human skin. Such ‘intelligent’ textiles can be actually regarded as an efficient delivery system for cosmetic or pharmaceutically-relevant substances through their contact with the skin [1].

The use of fabrics and garments as a tool for a powerful delivery of healthy substances on human skin is an old concept belonging to the philosophy of the Ayurvedic medicine, known as Ayurvastra: an ancient discipline elucidating the beneficial properties of extracts of natural herbs [2]. Over the last decade, functional textiles have tremendously evolved thanks to the active cooperation of chemists, physicians, and textile engineers. Indeed, in parallel with the improvement of the already well-established doping and coating procedures, methods based on microencapsulation and grafting technologies have gained increasing popularity in the production process of these products, ensuring enhanced quality of the resulting vehicles [1,3,4,5].

In recent years, innovative ‘biofunctional’ applications have been receiving attention in the field of cosmetotextile technology, mostly to meet the needs of people with highly sensitive skin. These kinds of material are indeed becoming diffusely appreciated as an innovative vehicle for the local treatment of skin diseases caused by microbial agents [6,7] or sunlight induced photooxidation [5,8].

Aimed at limiting the toxicity and the high environmental impact of many synthetic molecules often used by the biofunctional textile manufacturers [6], extracts of vegetal and animal origin are becoming increasingly preferred even by big companies, as a natural source of biomolecules for their production. Indeed, it has been widely demonstrated that antioxidants from natural sources can sensitively attenuate the effects of reactive oxygen species (ROS), sometimes even more efficiently than endogenous antioxidants [9,10].

Flavonoids and phenolic acids are among the most important chemical classes exhibiting antioxidant activity, mostly via radical-scavenging mechanisms [8,11]. This evidence along with the their strong interactions with fiber material has fueled the interest by scientists towards the functionalization of textiles by these molecules [8,12,13]. In this context, natural dyes, extracted from various species of wild plants or crops, are particularly appreciated because, in addition to the aforementioned beneficial biofunctional properties, they are also able to confer peculiar soft colors with a unique shade. A precious source of colored bioactive molecules is represented by biomasses, which constitute waste material from agricultural and food processing industries. This practice is in perfect line with the “circular economy” fundamentals [14].

Among others, the dried outer skin of onions (*Allium cepa* L.), representing the waste of onion consumption and processing, contains a multitude of colored bioactive phenolics, some of which are absent in the edible part of the vegetable [15,16,17]. In depth investigations at a molecular level have revealed that quercetin and its glucosides are the most abundant phenolics in onion outer dry layers, which explains the strong antioxidant effects of the resulting extracts [18,19,20].

In this paper, the waste of onion skin from the ‘Dorata di Parma’ cultivar was used as a new source of bioactive compounds to produce wool yarn with antioxidant and UV-protection properties. Besides its functional potential, this kind of biomass was chosen by the authors for many other reasons. First of all, onion is the second most important horticultural crop after tomatoes with a worldwide current production of around 98 million tons [21], making its related by-products widespread and easily available. Furthermore, onion waste is an easy handling biomass because of its low weight and dryness state, and has been historically used in Europe and Arab countries for dyeing textiles with deep yellow and orange nuances [22]. The exploitation of this biomass for the production of biofunctional textiles therefore represents an intriguing link between tradition and innovation.

## 2. Results and Discussion

**Figure 1:**
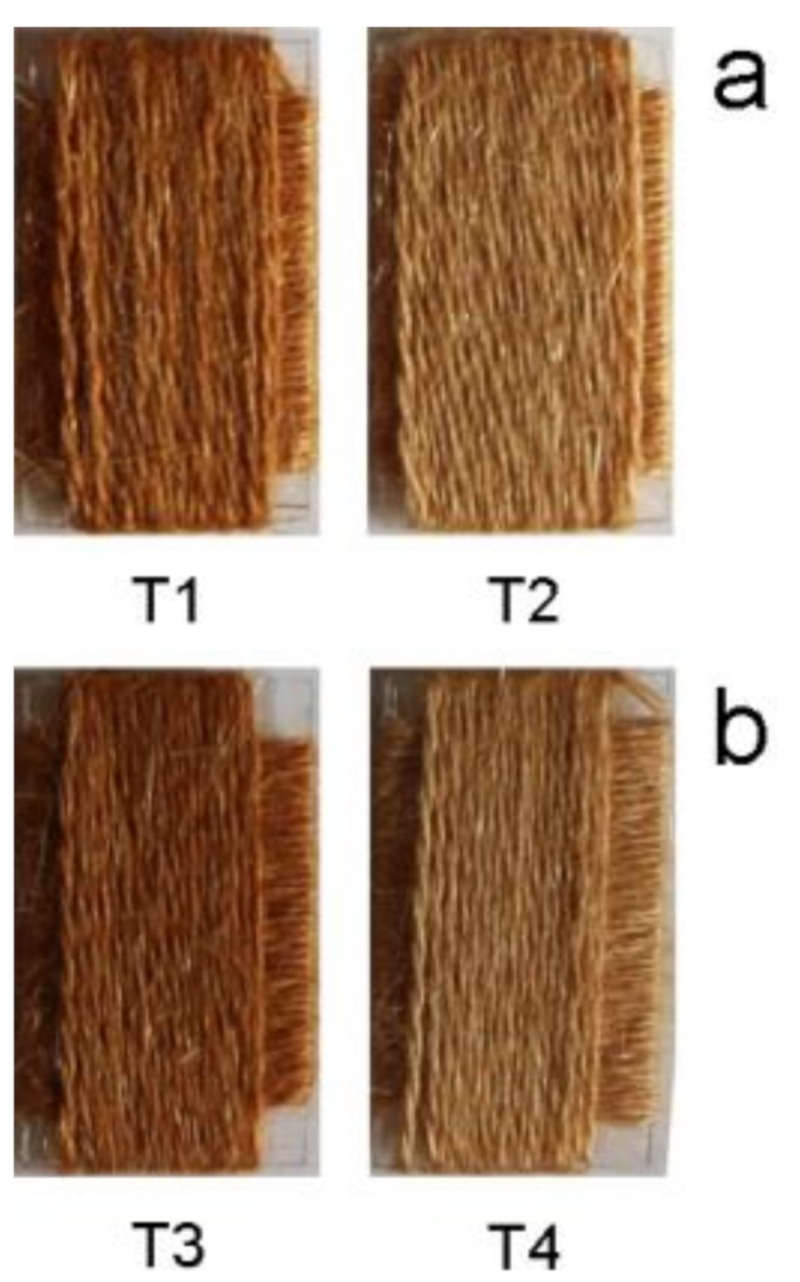
Wool yarn dyed with different percentage of onion skin (**a**: 100%, sample T1 and **b**: 75%, sample T3) with respect to the dry wool weight. Sample T2 and T4 were dyed in the residue bath previously used to dye samples T1 and T3, respectively.

Biomolecules from dried onion skins were extracted through a traditional boiling in H_2_O (see Material and Methods section for details) and readily used for dyeing scoured wool skeins in two successive dyeing cycles and using two different percentage (100% and 75%) of onion skin with respect to the dry wool weight (Figure 1). The use of chemicals was deliberately avoided in both the extraction and dyeing processes. Instead, the application of mordants was intentionally circumvented in order to favor the slow release of molecules from the fibers once they are in contact with the human skin. A multi-analytical approach, based on chromatographic techniques and a variety of conventional and reflectance UV–vis spectroscopic methodologies, was utilized to characterize the optical properties, the chemical composition and the biofunctional behavior of the aqueous extract and textiles. Moreover, the water extract was also tested in a cell-based assay to verify its safety and exclude any toxicity, such as the induction of proapoptotic effects.

### 2.1. Optical Properties of Skin Extracts and Cosmeto-Textiles

**Scheme 1:**
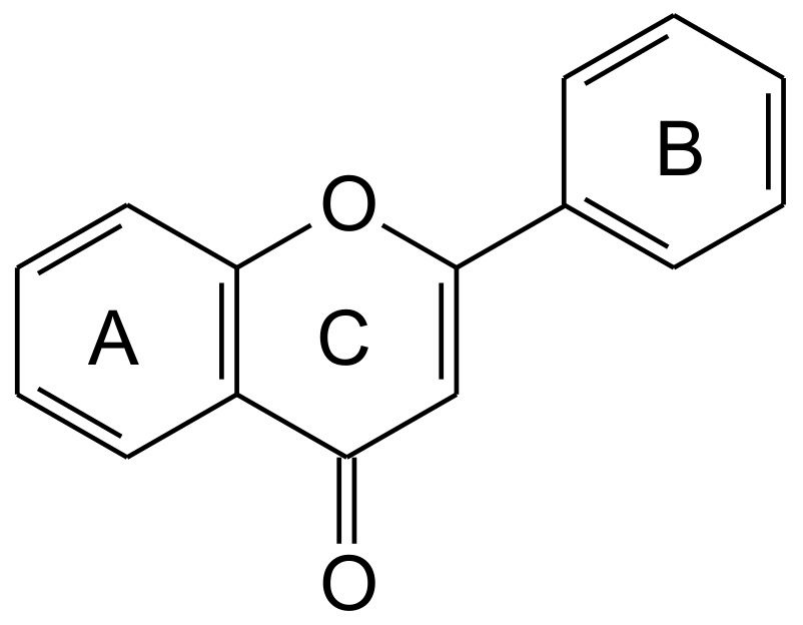
General structure of flavones.

**Figure 2:**
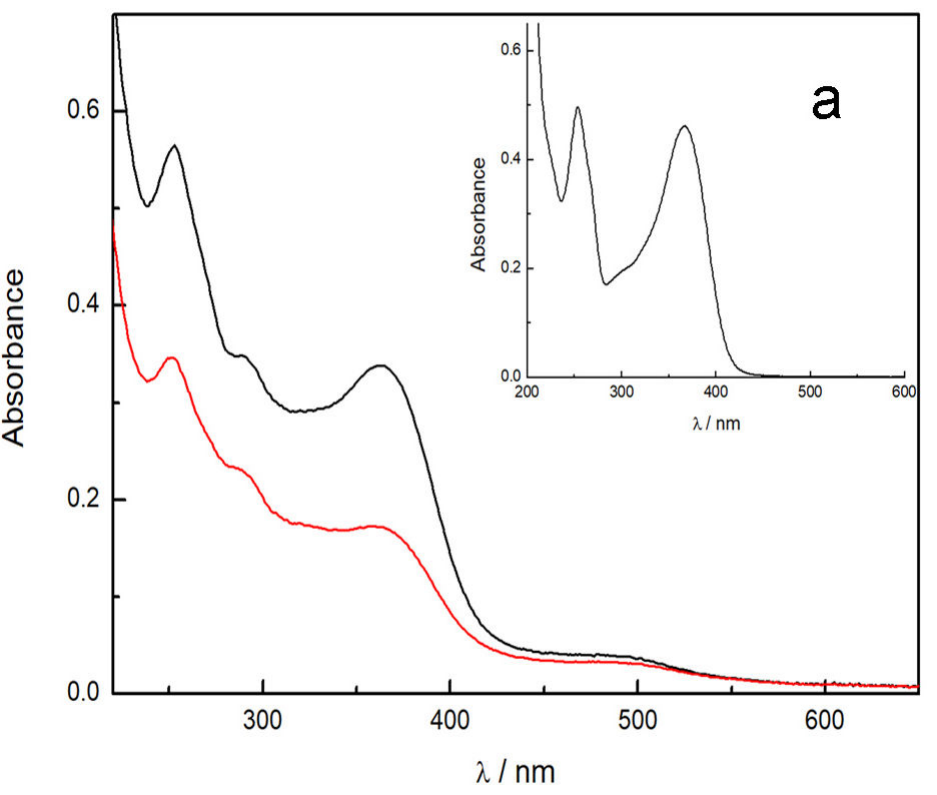

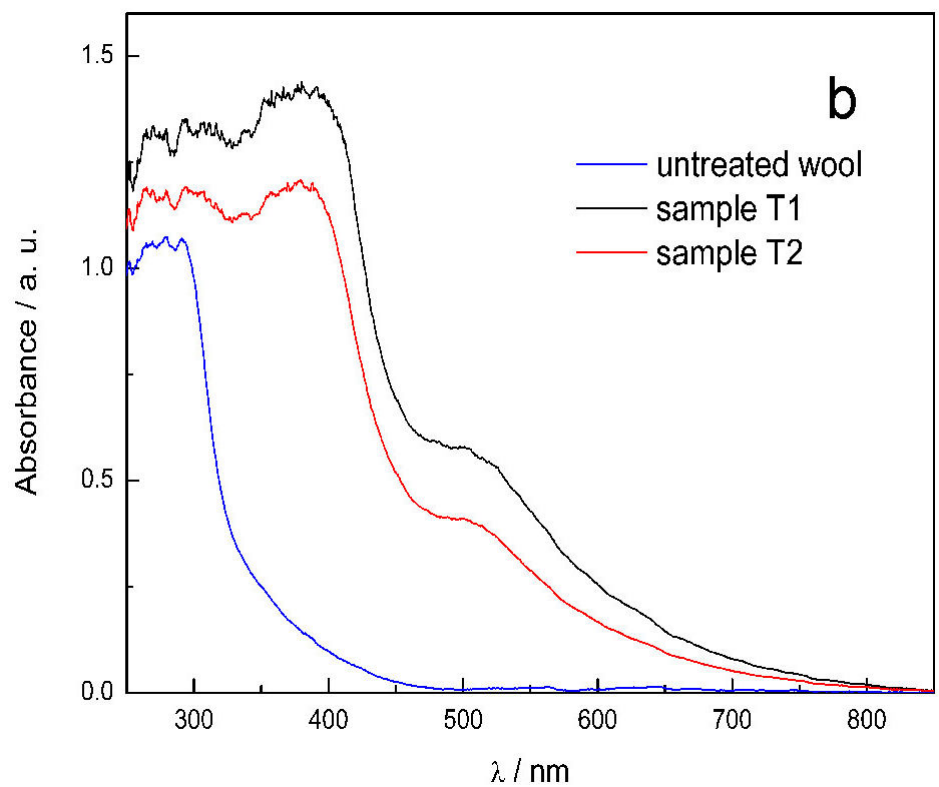
(**a**) Absorption spectrum of onion skin aqueous extract before (black) and after (red) the first dyeing cycle (100-fold diluted with H_2_O). Insert: absorption spectrum of quercetin in water. (**b**) Absorption spectra of untreated wool (UW, blue) and dyed wool in onion skin aqueous extract (T1, black) and its residue (T2).

The absorption spectrum of the aqueous onion skin extract (Figure 2a) shows the typical flavonoid features (Scheme 1) consisting of a band with maximum at 360 nm (Band I), assignable to the cinnamoyl system formed by B and C rings, a weak signal at 290 nm (Band II) attributed to the A and C ring benzoyl moiety and a band centered at 250 nm ascribable to the C ring [23]. A further band is observed at about 495 nm, probably due to the formation of flavonoid aggregates or to flavonoid degradation products. In particular, the absorption profile corresponds well with that of quercetin, as expected being quercetin and their glycoside derivatives the main flavonoid components found in outer dry onion skin [24].

This result is confirmed by the HPLC-MS and MS^2^ analysis (Section 2.2). The absorption maxima retrieved on all textile samples are slightly shifted to longer wavelengths with respect to those observed for the aqueous extract (Figure 2b), likely because of a specific interaction of flavonoid dyes with wool. The samples dyed with the original onion skin water extract (T1 and T3) showed a deep red-brown color, whereas those dyed in the residue dyeing solution (T2 and T4) exhibited a pale brown color, as indicated by positive values of a * and b * colorimetric coordinates (Table 1). The color (Figure 1) is mainly determined by the tail of the flavonoid absorption Band I, centered at 380 nm, and the bathochromic unidentified component at 510 nm (Figure 2b). Lower absorbance and colorimetric index values are obtained for samples T2 and T4, that were dyed with the residue solution used to prepare samples T1 and T3, respectively, indicating a lower dye uptake on wool fibers. This result was anticipated by the drastic decrease of absorbance detected for the residue dyeing solution (Figure 2a). On the other hand, no sensitive variation of color parameters and absorption features were observed when the onion waste amount was reduced from 100% to 75% with respect to the wool weight. This indicates that the dye accumulation on wool fibers is equivalent. Very interestingly, the spectra of all textile samples, even those dyed in a less concentrated onion skin extract (T3), showed absorption values higher than the untreated wool in all the UV–vis spectral range including the UVa and UVb (Figure 2b). This finding highlights the potential protection function of the prepared textile from the photo-oxidative damage caused by UV radiations on human cell skin as already reported for other natural dyes [25,26].

### 2.2. Metabolite Profiling of Polyphenols in Onion Extracts

Several flavonols have been described in onions, with quercetin derivatives being the most abundant ones [27]. The glycosyl moiety of these quercetin derivatives is almost exclusively glucose, which is linked to the 4′, 3, and/or 7-positions of the aglycone [27]. In particular, quercetin-4′-glucoside and quercetin 3,4′-diglucoside have been frequently found in the literature as the main flavonols present in onion extracts [27]. Derivatives of kaempferol and isorhamnetin have also been detected as minor flavonols [27]. Some studies have reported that small yellow onion bulbs and aerial parts contain several phenolic acids [28]. Red onion anthocyanins are mainly cyanidin glucosides acylated with malonic acid or non-acylated [27]. Some of these compounds present unique structural features, like 4′-glycosylation and unusual substitution patterns of sugar moieties [27].

**Figure 3:**
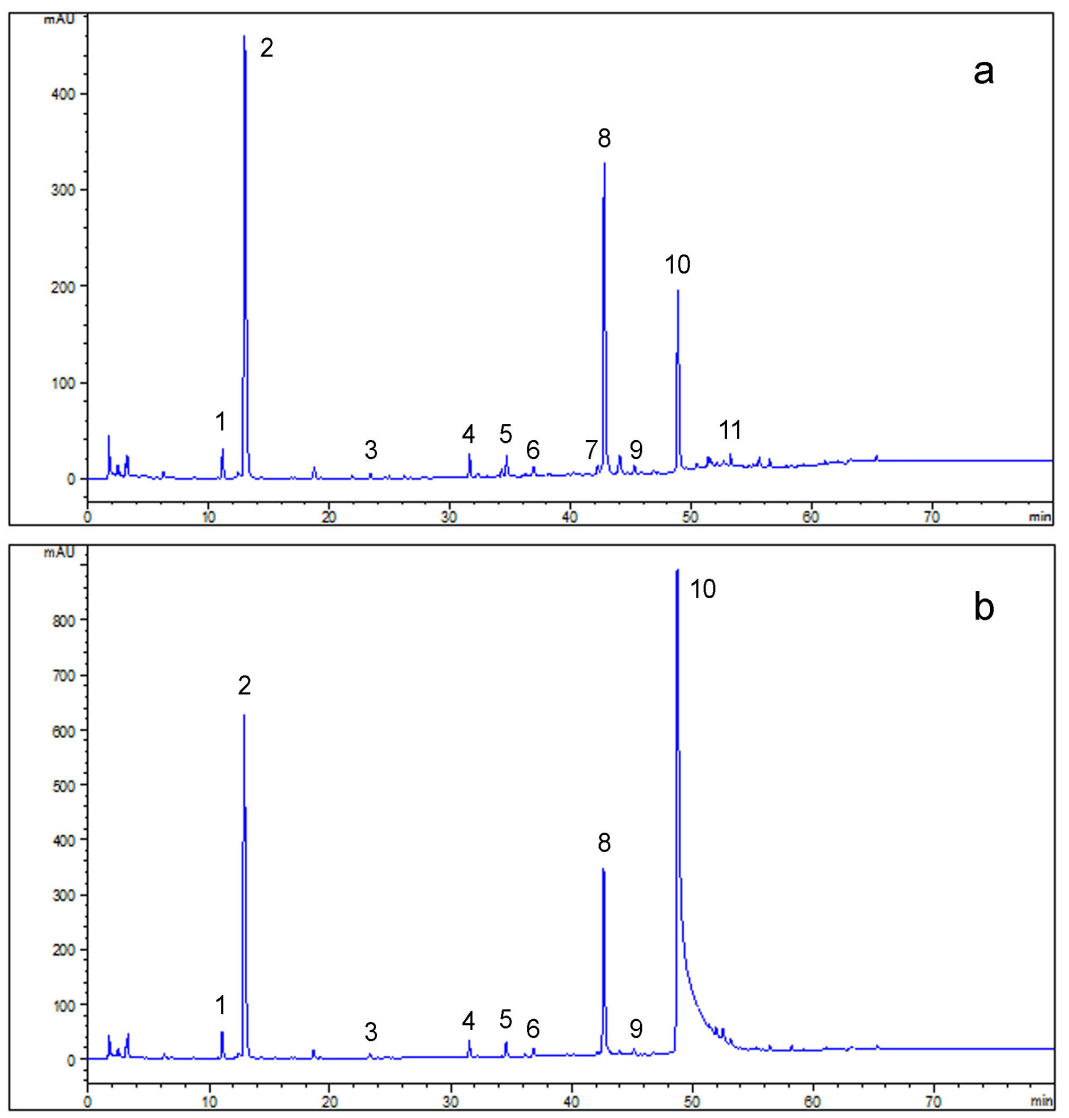
Chromatogram acquired by HPLC-UV/DAD analysis of the onion water extract at 254 nm before (**a**) and after (**b**) the first dyeing cycle. For peak identification, see Table 2.

As regards the extracts from the ‘Dorata di Parma’ onion skin analyzed in this work, UV–vis data, together with MS and MS^2^, allowed us to identify 11 compounds in the aqueous extract, belonging to phenolic acids and flavonols (Table 2). The glycosylation sites of quercetin glucosides and diglucosides was tentatively assigned in this study on the basis of the literature data [24,29,30,31]. Protocatechuic acid was found to be the main phenolic acid, while quercetin-4′-glucoside and quercetin were the major peaks for flavonols (Figure 3a). The same trend was observed for the chromatograms obtained for the residues after dyeing (Figure 3b). A previous work on onion solid wastes has shown a similar phenolic profile [17].

However, many studies have described quercetin 3,4′-diglucoside and quercetin 4′-glucoside as the main flavonols present in onion extracts [24,29,30,31]. The different chromatographic profiles obtained in the present work for onion extract and residues after dyeing may be attributed to the hydrolysis of quercetin 3,4′-diglucoside and quercetin 4′-glucoside, due to the high temperature applied during the extraction and dyeing procedures in the aqueous medium. As regards protocatechuic acid, it represents the main oxidation product of quercetin in the presence of air, as previously described in the literature [32], and it is therefore a product of quercetin decomposition under the conditions applied during the extraction. Protocatechuic acid is produced by the oxidation of quercetin during the process of browning [33,34]; it can also be further oxidized to protocatechuquinone [35]. Protocatechuic acid has been found in the outer scales of yellow onion bulbs, where it is known to exert an antifungal activity [33]. It should be specified that the release of quercetin from its glucosides by hydrolysis has been described to proceed faster than quercetin oxidative decomposition in onion solid wastes [17].

### 2.3. Measurement of Total Phenolic Content (TPC)

The amount of total phenolic compounds was determined spectrophotometrically by applying the original Folin–Ciocalteu method with only few modifications [36].

In the present study, the Folin–Ciocalteu method was applied both to the diluted dry onion skin extract and to the hydro-alcoholic solutions containing the differently treated textiles (Table 3). In order to simulate as closely as possible the delivery process of phenols to human skin, the assay for textiles was performed on the intact fiber, thus avoiding aggressive treatments which can favor an extreme and disproportionate biomolecule release. The test was also performed on the untreated wool (UW), and the result obtained was in agreement with the well documented, nonspecific, oxidation ability of the Folin–Ciocalteu reagent towards non-phenolic organic as well as inorganic substances [37,38]. Indeed, it has been demonstrated that reducing agents, including certain amino acids, can interfere with the spectrophotometric assay, leading to an overestimation of the phenolic content [37,38]. We assume this value (referred to as sample UW in Table 3) originated from specific amino acids sequences embedded in the keratin wool [39]. However, this is not a serious concern in the present study, since the same test was performed for comparative purposes on differently treated textiles.

In line with our expectations, the phenol loading abruptly decreased in successive dying cycles (samples T1 and T2, Table 3). This result unambiguously finds its roots in the lower total phenol content in the dying bath after the first dying cycle. Accordingly, we estimated that about 40% (30.85 ± 0.57 mg Eq. GA/g onion skin; with GA standing for gallic acid) of the initial phenol content was still present in solution after the first dipping step. This evidence is further supported by the consistent decrease of absorbance of the flavonoid feature (360 nm) observed in the dyeing solution after the first dyeing step (Figure 2a), that consequently led to a lower dye uptake (Figure 2b) for the sample within it dyed (T2, second dyeing cycle). Interestingly, we found that using a slightly lower amount of onion skin in the preparation of the dying solution (75% of that previously described) did not significantly alter the entity of adsorbed phenolic species onto the wool textile (samples T3 and T4, Table 3). This observation is consistent with the almost identical colorimetric coordinate values (Table 1) and absorption spectra (data not shown) of T1 and T3 samples.

### 2.4. Determination of Total Antioxidant Capacity (TAC) of Onion Skin Extracts and Biofunctional Textiles

A single method to univocally determine the TAC of a plant extract does not actually exist. Moreover, although numerous methods are currently in use for TAC estimation, standardized protocols are still far from being officially approved. The availability of official protocols is furthermore complicated by the common practice of researchers to adapt, case-by-case, procedures already developed by other authors for specific applications. In the study of complex matrices, it is of crucial importance for researchers to take in mind that every method is selective for specific antioxidant families and reactions [40]. Therefore, in order to evaluate the TAC of a phytocomplex, the results from different methods have to be cumulatively considered. In this context, both UV–vis spectrophotometric and spectrofluorimetric-based assays can provide important complementary information. Accordingly, in the present study, TAC was appraised on the same samples by relying upon the ferric reducing antioxidant power (FRAP) spectrophotometric method as well as the spectrofluorimetric-based oxygen radical absorbance capacity (ORAC) assay.

Basically, two different reaction mechanisms can be exploited for TAC measurements: one based on a hydrogen atom transfer (HAT) process, the other on a single electron transfer (ET) [38,41]. It is worth highlighting that some polyphenol sub-families respond better to HAT-based assays, while others respond better to ET-based ones.

ORAC is a ‘pure’ HAT-based method that measures the ability of an antioxidant (or a mixture of antioxidants) to quench free radicals by means of hydrogen donation. In the ORAC assay, the pool of antioxidants in the phytocomplex and the selected substrate (i.e., fluorescein in the present study) compete for thermally generated peroxyl radicals [37,42]. The peroxyl radical is generated by thermal decomposition of 2,2′-azobis(2-methylpropionamidine)dihydrochloride (AAPH) and preferably abstracts a H^·^ from the antioxidants in the phytocomplex [41]. As a consequence, its reaction with the fluorescein probe occurs later. Peroxyl radicals decrease the fluorescence of the probe, and the decrease constant for its fluorescence decay profile is used to measure the antioxidant capacity of the sample under investigation [37,41,42,43,44]. Unlike many methods for TAC determinations (including FRAP), the ORAC assay holds the big advantage to mimic physiologically relevant perturbations.

Previous studies by other authors have clearly highlighted a distinct difference in the ORAC values from hydrophilic and lipophilic extracts of fruits and vegetables [43,44]. Most of the time, hydrophilic ORAC (H-ORAC) values were higher than those generated by lipophilic extracts (lipophilic ORAC, L-ORAC) [43,44].

Contrary to ORAC, the FRAP method is exclusively based on an ET mechanism. Indeed, FRAP assay measures the ability of antioxidants to reduce the Fe^3+^-TPTZ (with TPTZ standing for 2,4,6-tris(2-pyridyl)-s-triazine) complex to its ferrous form (Fe^2+^-TPTZ) at low pH [37]. It has been reported that the reducing power of polyphenols measured through the FRAP test is somehow related to the degree of hydroxylation and to the extent of conjugation in their molecular structure [38,45].

As a consequence of its underlying mechanism, FRAP cannot be used for the analysis of compounds acting as radical quenchers via HAT process. However, this is only an apparent drawback of the method, since, in combination with other assays, it can be very useful in distinguishing dominant mechanisms by different antioxidants [37], and, in turn, establishing the molecular species contributing to TAC. Instead, the absence of an oxidable substrate in the FRAP analysis is a serious limitation of the method since it does not allow evaluating the potential protective properties of the antioxidant pool in the extract. From data listed in Table 3, a strict correlation (R^2^ = 0.88) between data from the FRAP and ORAC assays readily comes to light. These results unambiguously suggest that the chemical nature of the functional molecules in the onion extract does not influence the degree of adsorption onto the wool fiber. A remarkably significant (R^2^ = 0.98) relationship between results from FRAP and Folin–Ciocalteu tests also emerges, thus supporting the opinion of some authors [46] that the former method is actually applicable to measure the total antioxidant concentration of a mixture. On the other hand, although the Folin–Ciocalteu assay is diffusely used to measure the total phenolics in natural products, its basic mechanism relies upon an ET process and, therefore, it can be considered another method for TAC estimation. The conventional Folin–Ciocalteu reagent has proven to be only applicable to water-soluble antioxidants [37,38].

Trying to summarize, on the basis of all the above, one can state that the higher the loading of the phenol constituents onto the textile, the greater the TAC of the adsorbed extract.

### 2.5. Evaluation of TPC on Strip after Percutaneous Absorption

The Folin–Ciocalteu assay was applied for the evaluation of the amount of phenolic species transferred from textiles to skin. Biofunctional textiles containing phenol species were fixed on forearms of two volunteers (named to as A and B) for periods of 12 and 24 h. The outermost layers of the stratum corneum (SC) were then obtained using strips.

**Figure 4:**
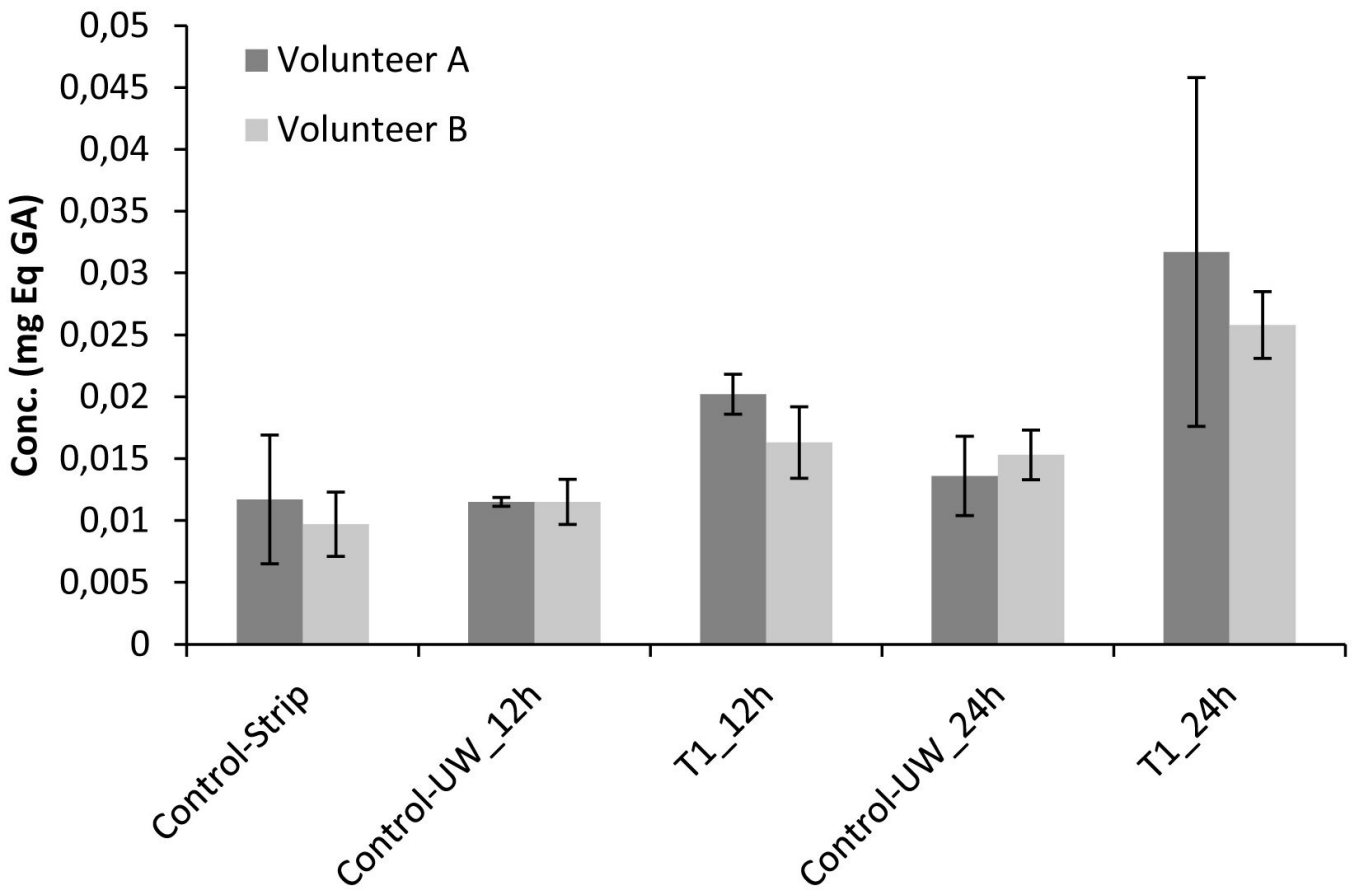
Values expressed as mg Eq. GA obtained from control, control-textiles and biofunctional textiles after 12 and 24 h of application onto the skin of the two healthy volunteers. UW is for ‘untreated wool’; T1 refers to Textile #1. All analyses were performed in triplicate.

The results obtained, expressed as concentration (mg Eq GA) are shown in the plot in Figure 4. The bar couples related to biofunctional textiles (i.e., 3 and 5 in Figure 4) are always higher than those corresponding to controls (i.e., 1, 2, and 4). This result further demonstrates that phenols are able to efficiently pass from textile to skin.

### 2.6. Determination of Lipoperoxidation by 2- thiobarbituric acid (TBA) Assay

Skin lipids can undergo oxidative damage as to UV ray exposure. The entity of lipid peroxidation can be evaluated in several ways, including the quantitative analysis of end products of the photooxidative process. Malonaldehyde *bis*(dimethyl acetal) (MDA) is one of such end products, and its quantification as thiobarbituric derivative is often used to quantify the extent of lipid peroxidation in an injured tissue [47].

The reaction between TBA and MDA yields an intensely colored chromogen fluorescent red adduct [48] that can be easily analyzed spectrophotometrically.

In this work, the strips were irradiated with UV light spectrally simulating solar radiation, and lipid peroxides (LPOs) removed from the SC were measured by TBA assay. The results obtained with TBA test were expressed as MDA equivalents (µM MDA) using a regression curve for pure MDA-TBA complexes. As indicated by the elevated R^2^ value (>0.98), the method is significantly linear within a rather broad concentration range, namely, 0.05–1 µM of MDA.

**Figure 5:**
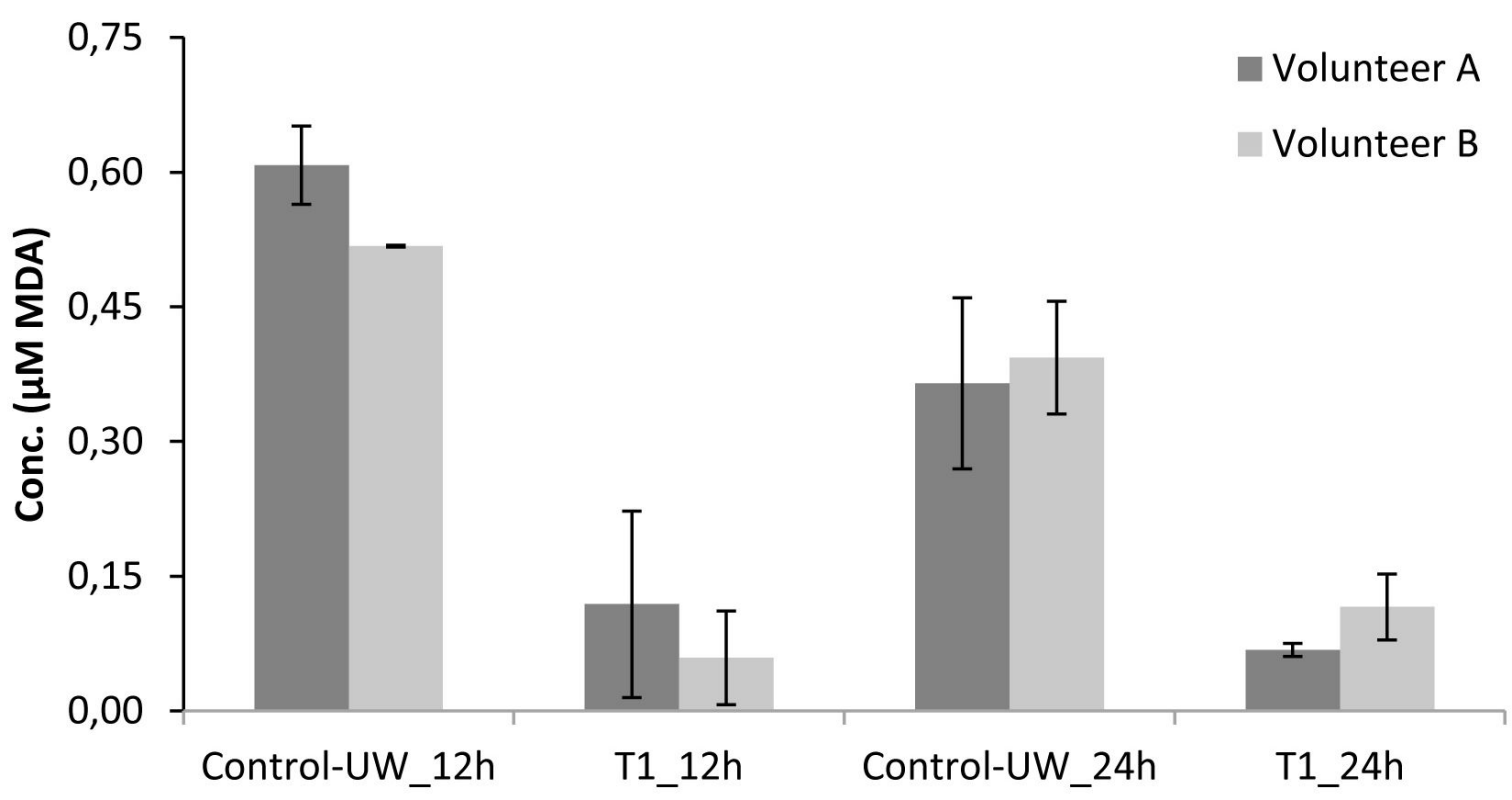
Concentration (µM) values after UV irradiation of the strips obtained from control, control-texiles, and biofunctional textiles after 12 and 24 h of application onto the skin of the two healthy volunteers. UW is for ‘untreated wool’; T1 refers to Textile # 1. All analyses were performed in triplicate.

In Figure 5 the values expressed as concentration of MDA (µM) obtained for strips irradiated with UV radiation are shown. Comparing the results obtained for control-textiles (UW) and biofunctional textiles (T1), a lower concentration of MDA was always detected after the application of the treated textiles.

The results obtained in the present study clearly indicate that a certain quantity of antioxidant has been released from biofunctional textiles to SC. Moreover, these compounds were able to protect skin against lipid peroxidation following UV radiation. This finding agrees with the greatest capacity (highest absorbance values) of dyed yarns to absorb UVa and UVb radiation with respect to untreated wool (Figure 2b). As evident from data in Table 4, the obtained values of the LPO inhibition (%) were quite similar for both volunteers. Being aware of the limited number of recruited volunteers, we have here highlighted the potential of the produced material as biofunctional textiles.

### 2.7. Analysis of Cell Viability and Induction of Apoptosis after Treatment of RAW 264.7 Cells with Onion Aqueous Extract

**Figure 6:**
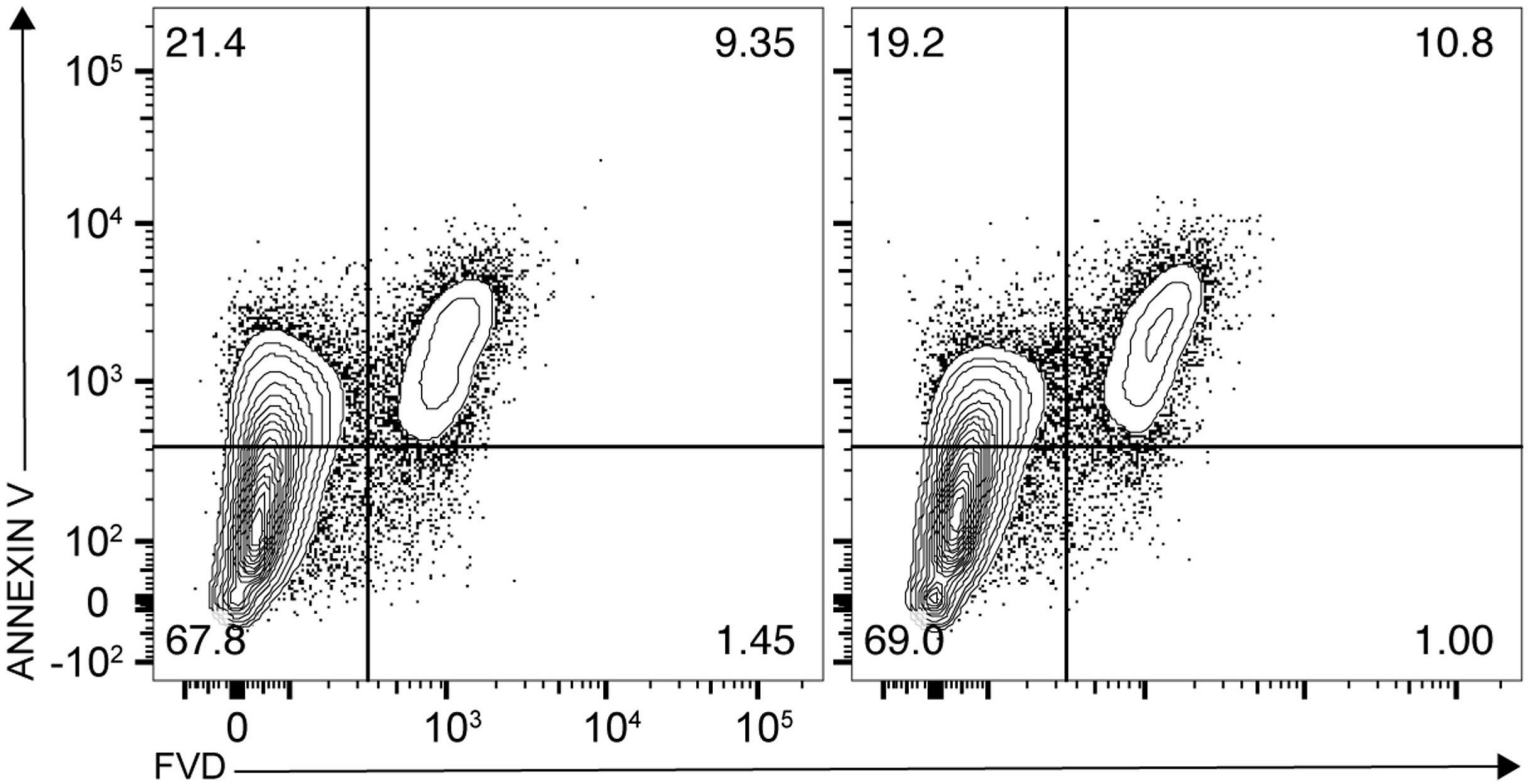
Percentage of living and apoptotic RAW 264.7 macrophages, untreated (**a**), or exposed for 24 h to 80 µg Eq. of GA/mL (**b**). The percentage of living cells (67.8% and 69.0% respectively) early apoptotic cells (21.4% and 19.2%) and late apoptotic cells (9.35% and 10.8%) is not significantly different in untreated cells and cells treated with extract from onion skin.

The safety of the aqueous extract from onion skin was investigated in RAW 264.7 cells, a macrophage cell line commonly used to assess biologically active compounds for their toxicity and/or effects on inflammatory processes and immune responses. Macrophages were untreated or treated for 24 h with a single dose of onion extract, corresponding to 80 µg Eq. of GA/mL. Cell viability and induction of apoptosis were then assessed by citofluorimetric analysis, after staining the cells with a fluorochrome conjugated Annexin V, a protein commonly used to detect apoptotic cells. Annexin V is an endogenous human protein endowed with a high affinity for membrane-bound phosphatidylserine (PS). The number of annexin V binding sites per cell with the onset of apoptosis increases 100-to 1,000-fold during apoptosis. PS exposure on the cell surface occurs before DNA fragmentation, therefore annexin V is a sensitive marker of the early to intermediate phases of apoptosis [49]. The results, shown in Figure 6, demonstrate that, as expected, aqueous extracts of onion skin do not have a significative influence on cell viability and they did not induce a proapoptotic effect, further confirming the safety and the absence of cellular toxic effects of the polyphenolic compounds found in the extract.

## 3. Materials and Methods

### 3.1. Chemicals and Reagents

Folin–Ciocalteu reagent, 2,4,6-tris(2-pyridyl)-s-triazine (TPTZ), 6-hydroxy-2,5,7,8-tetramethyl-2-carboxylic acid (Trolox), 2,2′-azobis(2-methylpropionamidine)dihydrochloride (AAPH), hydrochloric acid (HCl), ferric chloride (FeCl_3_), sodium acetate (NaOAc), sodium carbonate (Na_2_CO_3_), gallic acid (GA), HPLC grade methanol (MeOH), acetonitrile (ACN), phosphate buffered saline (PBS), fluorescein sodium salt, malonaldehyde bis(dimethyl acetal) (MDA), 2-thiobarbituric acid (TBA), and trichloroacetic acid (TCA) were purchased from Sigma-Aldrich (Milano, Italy). Protocatechuic acid, vanillic acid, ellagic acid, and quercetin were purchased from Fluka (Milan, Italy). Isorhamnetin was from Roth (Karlsruhe, Germany). Formic acid (HCOOH) and methanol (MeOH) were from VWR (Milan, Italy). Water was purified by using a Milli-Q Plus185 system from Millipore (Milford, MA, USA). Corneofix^®^ strips were purchased from G.F. Secchi (Como, Italy). PBS solution was prepared as follows: dibasic sodium phosphate (Na_2_HPO_4_,15.50 g) was dissolved in distilled water (840 mL), and monobasic potassium phosphate (KH_2_PO_4_, 1.52 g) was dissolved in distilled water (160 mL); these two solutions were then mixed and pH was fixed at 7.6.

Dry onion skins from the ‘Dorata di Parma’ cultivar were kindly provided by Cannara Onion Producers’ Union (ConsorziodeiProduttoridellaCipolla di Cannara, Italy) and processed without further pre-treatments. The raw wool yarn used in this study was provided by the cultural association ‘Franco Brunello’ (Enego, Italy). The Brij^®^S10 non-ionic surfactant used for the scouring of wool yarn was purchased from Sigma-Aldrich (Milano, Italy).

For the cellular assays, Trypsin-EDTA was purchased from Thermo Fisher Scientific (Trypsin-EDTA, phenol red, Cat# 25300062), Fixable Viability Dye eFluor^®^ 780 kit was from eBioscience, (S. Diego, CA, USA) as well as PerCP-eFluor 710-conjugated Annexin-V kit. Flow cytometry analyses were performed using a LSR Fortessa Flow Cytometer (BD Biosciences, San Jose, CA, USA) and data were analyzed using FloJo software (version 10.5.3, FlowJo LLC, Ashland, OR, USA).

### 3.2. Photophysical Measurements

Folin–Ciocalteu as well as FRAP tests were carried out at 25 °C with a Varian Cary 100 (Varian Inc., Palo Alto, CA, USA) dual beam, dual chopper spectrophotometer. Emission intensity for ORAC experiments were determined by a HORIBA Scientific FluoroMax^®^-4P spectrofluorimeter (Kyoto, Japan) operated by FluorEssence^TM^. The instrument provides corrected fluorescence emission spectra taking into account both the monochromator response and detector sensitivity.

Reflectance spectra on solid samples were performed using a portable instrument composed of Avantes parts and equipped with a quartz fiber optic system already described in a previous paper [50]. The reflected light was collected under a 21° angle respect to the surface, using a 0/0 geometry, thus avoiding the specular reflected light. A 99% Spectralon^®^ diffuse reflectance standard (Labsphere, North Sutton, NH, USA) was used for calibration. The instrument provides a 8 nm spectral resolution, whereas the fiber optic probe allows a surface area of 2 mm^2^ to be analyzed. The reflectance spectra were expressed in terms of pseudoabsorbance, A′(λ), according to Equation (1) [51]

A′(λ) = log [1/(0.01·R(λ))](1)

where R is the measured reflectance at each specific wavelength λ. The color of dyed wool samples was assessed in terms of CIELab values. In the CIELab color space the color is defined by three numerical coordinates: L * for the lightness and a * and b * for the green–red and blue–yellow color components, respectively. The lightness value, L *, ranges from L * = 0 for the darkest black to L * = 100 for the brightest white. The a* coordinate represents the green–red component, with green in the negative direction (a * < 0) and red in the positive direction (a * > 0). The b * coordinate indicates the blue–yellow component, with blue in the negative direction (b * < 0) and yellow in the positive direction (b * > 0). Colorimetric measurements were carried out on a Konica Minolta CM-700d spectrophotometer (Tokyo, Japan) under D65 illuminant and 10° standard observer. Four measurements were made for each sample recording the percentage reflectance values over the 350–750 nm spectral range.

### 3.3. Extraction of Polyphenols from Dried Outer Onion Skin

A 200 mL volume of H_2_O was added to 2 g of onion skin and the resulting solution was kept under magnetic stirring at 95 °C for 1 h. For an efficient removal of onion skin debris, the extract was filtered through paper filter. Extraction was performed in triplicate. The extracts were stored at −20 °C until further analysis. The same extraction procedure was followed to prepare the dye bath for wool dyeing experiments.

### 3.4. Scouring and Dyeing Procedure

The wool yarn was washed with a Brij^®^S10 non-ionic detergent solution (5 gr/L, yarn to liquid ratio 1:100 *w*/*v*) at 50 °C for 30 min in order to remove eventual impurities due to the manufacturing processes thus improving its hydrophilicity. The scoured wool was then rinsed several times with H_2_O and air-dried at room temperature for 24 h. This sample will be referred to as untreated wool (UW) along the text.

Wool dyeing was carried out by the exhaustion method using a 1:80 (*w*/*v*) yarn to liquid ratio and different percentage of onion skin (100% and 75%) with respect to the dry wool weight. Sample #1 (T1) was obtained by dipping 1 g of scoured wool into 80 mL of onion skin aqueous extract (100% onion skin with respect to the dry wool weight) prepared as described above and previously heated at 40 °C. The temperature was then increased to 95 °C and kept constant for 60 min. The wool was then removed, rinsed several times with H_2_O until the aqueous phase appeared uncolored and air dried in the dark at room temperature. Sample #2 (T2) was obtained by dipping 1 g of scoured wool in the dyeing solution residue previously used for the preparation of T1 and dyed under the same experimental conditions used for T1. Samples #3 (T3) and #4 (T4) were obtained following the same procedure used for T1 and T2 respectively, in a less concentrated onion skin aqueous extract (75% onion skin with respect to the dry wool weight). For each sample, the dyed wool yarn was carefully rolled around a Plexiglas sheet in order to have a 3 × 1 cm skein with a flat and homogeneous surface therefore adequate for in situ reflectance and colorimetric measurements.

### 3.5. HPLC-UV/DAD Analysis of Polyphenols

HPLC analyses were performed on an Agilent Technologies (Waldbronn, Germany) modular model 1100 system, consisting of a vacuum degasser, a quaternary pump, an autosampler, a thermostated column compartment and a diode array detector (DAD). The chromatograms were recorded using an Agilent Chemstation for LC and LC-MS systems (Rev. B.01.03). The HPLC analysis of phenolic acids and flavonols was carried out on an Ascentis Express C_18_ column (150 × 4.6 mm I.D., 2.7 µm, Supelco, Bellefonte, PA, USA). The mobile phase was composed of 0.1% HCOOH (*v*/*v*) in both (A) H_2_O and (B) ACN. The gradient elution was modified as follows: 0–5 min 3% B, 5–45 min from 3% to 50% B, 45–60 min from 50% to 100% B, 60–80 min 100% B. The post-running time was 10 min. The flow rate was 0.8 mL/min. The column temperature was set at 25 °C. The sample injection volume was 10 µL. The UV/DAD chromatograms were acquired at 254, 330, 350, and 520 nm. Three injections were performed for each sample.

### 3.6. HPLC-ESI-MS and MS^2^ Analysis of Polyphenols

HPLC-ESI-MS and MS^2^ analyses were carried out using an Agilent Technologies modular 1200 system (Agilent, Waldbronn, Germany), equipped with a vacuum degasser, a binary pump, a thermostated autosampler, a thermostated column compartment and a 6310A ion trap mass analyzer with an ESI ion source. The HPLC column and the applied chromatographic conditions were the same as reported for the HPLC-UV/DAD system. The flow rate was split 2:1 before the ESI source.

The HPLC-ESI-MS system was operated both in the positive and in the negative ion modes. The experimental parameters were set as follows: the capillary voltage was 3.5 kV, the nebulizer (N_2_) pressure was 32 psi, the drying gas temperature was 350 °C, the drying gas flow was 11 L/min and the skimmer voltage was 40 V. Data were acquired by Agilent 6300 Series Ion Trap LC/MS system software (version 6.2, Agilent Technologies, Waldbronn, Germany). The mass spectrometer was operated in the full-scan mode in the *m*/*z* range 100–1000. MS^2^ spectra were automatically performed with helium as the collision gas in the *m*/*z* range 50–1000 with the SmartFrag function.

### 3.7. Determination of Total Phenol Content (TPC) by the Folin–Ciocalteu Method

The total phenol content (TPC) of each extract was determined in triplicate according to the Folin–Ciocalteu method described in [36] with only few modifications.

The Folin–Ciocalteu reagent was diluted 10-fold with H_2_O. A definite volume of extract (0.1 mL) was mixed with 0.75 mL of the diluted Folin–Ciocalteu reagent and incubated in the dark for 10 min at room temperature. The term ‘extract’ is related either to the onion extract 4-fold diluted with H_2_O or to the yarn sample (about 2.0 mg) added to a 0.1 mL of H_2_O/MeOH (1:1, *v*/*v*) solution. Then, 0.75 mL of 2% sodium carbonate (*w*/*v*) aqueous solution were added. The mixture was kept in the dark for 3 h before measuring the absorbance at 765 nm. The content of total phenolics was determined by using a standard curve prepared with gallic acid (GA) solutions previously treated in the same way as for the real samples. Therefore, results were expressed as mg of GA equivalents/g onion skin and as mg of GA equivalents/g textile.

The amount of phenolic species transferred from textiles to skin was also determined by the Folin–Ciocalteu assay. Each penetration area was stripped twice with two different strips which were then combined and immersed into a beaker containing the Folin–Ciocalteu solution.

### 3.8. Determination of the Total Antioxidant Capacity (TAC) by the FRAP Method

The reducing power was determined according to the method described in [36]. The FRAP reagent was prepared from 2.5 mL of a TPTZ solution (10 mM) in HCl (40 mM) and 2.5 mL of a FeCl_3_ aqueous solution (20 mM), mixed with 25 mL of NaOAc (300 mM, pH 3.6). For the determination of the antioxidant activity, 1.5 mL of FRAP reagent were mixed with 100 µL of H_2_O and 100 µL of the sample extract. The term ‘extract’ is related either to the onion extract 12-fold diluted with H_2_O or the yarn sample (about 2.0 mg) or the strip added to a 0.1 mL of H_2_O/MeOH (1:1, *v*/*v*) solution. The reaction mixture was allowed to stand for 4 min at room temperature before measuring the absorbance at 593 nm. Analyses were performed in triplicate for each sample. The total antioxidant capacity (TAC) values were determined from a calibration curve prepared with Trolox standard solutions, previously treated by applying the same procedure as for the real sample. The antioxidant capacity of the sample was expressed as mg of Trolox equivalents/g onion skin and as mg of Trolox equivalents/g textile.

### 3.9. Determination of the Oxygen Radical Absorbance Capacity by the ORAC Method

A slightly modified version of the method developed by Ou et al. was applied in this experiment [52,53].

A fluorescein solution (42 nM in PBS) freshly prepared each day was pre-incubated in a warm water bath (37 °C) for 15 min before measurements. AAPH (153 mM) was prepared daily by solubilizing 400 mg of AAPH in 10 mL PBS. In each cuvette, 2.25 mL of fluorescein and either 375 μL of sample (either the onion extract or 2 mg of fabric sample treated with 5 mL of PBS) or the same volume of blank (PBS) or of the standard solution (Trolox, 6, 12.5, 25, and 50 μM) were placed. Then, 375 μL of AAPH were added. The fluorescence was measured immediately after the AAPH addition and measurements were then made every 5 min until the relative fluorescence intensity was less than 5% of the value of the initial reading. The measurements were made in triplicate. Analyses were carried out on a spectrofluorimeter by fixing the excitation and emission wavelength at 493 nm and 515 nm, respectively. Both excitation and emission slits were set at 2.5 nm.

The ORAC final calculations were made by applying a regression equation built up with Trolox (used as standard) concentrations (*x*-axis) vs AUC (*y*-axis) values. AUC represents the area under the fluorescence decay curve, calculated as

(2) $$AUC\;=\;\left(5+\frac{f5}{f0}+\frac{f10}{f0}+\frac{f15}{f0}+\ldots \right)\times 5$$

where, f0 is the initial fluorescence intensity before AAPH addition at time 0, and f5, f10, f15 are the fluorescence intensity at time 5, 10, 15 min after the addition of AAPH. All the obtained AUC values were subtracted of that found blank.

Results were expressed as mg of Trolox equivalents/g onion skin and mg of Trolox equivalents/g textile.

### 3.10. Evaluation of the Percutaneous Absorption with the ‘Ex Vivo’ Stripping Method

The experimental protocol for the percutaneous absorption of antioxidant containing biofunctional textiles was conducted with two healthy Caucasian women volunteers (referred to as A and B in the following sections). The two volunteers, both aged 27 years, refrained to use any kind of cosmetics on their harms 14 days prior to the study. The biofunctional textile (wool dyed with onion skin extract, T1) and control textile (UW) were applied on subjects’ forearms by maintaining a close contact with the skin for time frames of 12 and 24 h as a bandage application. In order to avoid any passage of functional molecules from the functionalized textile and the compression bandage, a piece of impermeable paper was placed between the two compartments.

The skin penetration behavior was studied through the application of the stripping method as described in [10,54], with only few modifications. Corneofix^®^ strips were used for the stripping of the stratum corneum (SC) of each forearm. Each strip was applied to the skin surface and a hand pressure was exerted. After few seconds, the strip was removed in one gentle movement.

In order to compare the condition of SC after application of textiles with that of ‘untreated’ SC, also the stripping of the latter was evaluated and this sample was indicated as the ‘control’. Then the strips were submitted to two assays, i.e., Folin–Ciocalteu assay and TBA assay.

The protocol was approved by the Ethics Committee of Umbria Region and the study was conducted in accordance with the standards of Good Clinical Practice for trials of medicinal products in humans. Written informed consent was obtained.

### 3.11. Determination of Lipoperoxidation by Thiobarbituric Acid (TBA) Assay

In this study, the lipid peroxides (LPO) formed in the strip were measured by TBA assay. The strips were irradiated for 45 min using a light source simulating the UV solar radiation (1 × 10^5^ µW/cm^2^). Afterwards, the strips were immersed into a H_2_O/MeOH (1:1, *v*/*v*) solution (0.2 mL). Then, 1 mL of a solution made up with 0.4% (*w*/*v*) TBA and 15% (*w*/*v*) TCA in 100 mL of HCl solution (0.25 M) was added. The mixture was incubated for 1 h in a boiling water bath before measuring the absorbance at 534 nm with a UV–vis spectrophotometer.

Quantification of MDA-TBA complex was appraised by mean of a calibration curve prepared with MDA standard solutions at different concentrations in the same hydro-organic solvent as before.

The effect of phenol-containing biofunctionaltextiles on skin was also evaluated in terms of % LPO inhibition. This quantity was determined by applying the equation

(3) $$LPO\left(\%\;inhibition\right)\;=\;\left(1-\frac{\left[Ax-Ao\right]}{\left[Px-Po\right]}\right)\times 100$$

where, Ax and Ao represent the concentration values achieved by analyzing the strips submitted or not to UV irradiation, respectively. In this case, the strips removed the SC previously coated with phenol containing biofunctionaltextiles. Instead, Px and Po represent the concentration values achieved by analyzing the strips submitted or not to UV irradiation, respectively. In this case, the strips removed the SC previously coated with untreated wool. Once again, the subscripts “x” and “o” refer to irradiated or not irradiated sample, respectively. A value equal to 0% indicates that the antioxidant does not protect at all the skin against UV irradiation, while, a 100% of LPO inhibition means that the compounds released from textile have a complete antioxidant capacity.

### 3.12. Analysis of Cellular Viability and Apoptosis

RAW 264.7 cells were seeded into a 12 well plate at a density of 1.2 × 10^6^ cells/mL. The aqueous extract from onion skin was added to cells for 24 h at a final dilution of 1:10 (*v*/*v*), corresponding to a concentration of 80 µg Eq GA/mL. After the treatment, cells were harvested by trypsinization, washed with PBS and stained with Fixable Viability Dye eFluor^®^ 780 for 30 min. After washing with PBS and Flow Cytometry Staining Buffer, 5 μL of PerCP-eFluor 710-conjugated Annexin-V were added to cells and incubated at room temperature for 15 min. The percentage of PerCP-eFluor 710-positive apoptotic cells was determined by flow cytometry, and analyzed with FloJo software. Each experimental point was performed in triplicate.

## 4. Conclusions

In the present paper, we demonstrated that biofunctional wool textiles can be produced with waste material from agricultural and food processing industries. In particular, biofunctional textiles were efficiently realized by employing the dried outer skin of onions. This biomass holds several advantages, including its low weight and dryness state.

For the wool dyeing process, gold onion bulbs from the cultivar ‘Dorata di Parma’ were selected. Among the reasons accounting for this choice, the well-known rich content in polyphenols of this cultivar as well as the ability of its outer dried skin to confer peculiar soft colors with a unique shade are worth mentioning.

Textiles were produced with environmental-friendly manufacture practices without any addition of mordants. As a result, a material with a peculiar ability to protect skin against lipid peroxidation following UV radiation was manufactured in small scale. In order to draw a firm conclusion about the photoprotective action of the produced materials, additional studies on a larger number of volunteers are strictly required. Nevertheless, the results achieved in our investigation strongly suggest their high potential in protecting skin against lipid peroxidation following UV radiation.

The photoprotective and antioxidant activity of the biofunctional textiles under investigation were ascribed to the polyphenolic pool contained in the outer dried gold skins of onion. This assumption was confirmed by the application of a multi-analytical approach, based on the chromatographic, spectroscopic and spectrometric analysis of the aqueous extracts and the textile samples. Very importantly, citofluorimetric analysis demonstrated the safety and the absence of cellular toxic effects of the polyphenolic compounds present in the extracts.

This study lays the foundation for future investigations where other fibers and various waste material from agricultural and food processing industries will be evaluated for the production of smart textiles. In parallel, new dyeing modalities will be carefully considered with the aim to further enhance the loading and release of biofanctional molecules onto the selected material. Additionally, other properties, such as the antimicrobial activity of specific extracts, will also be deeply investigated.

## Figures and Tables

**Table 1:** Colorimetric coordinates of textile samples.

Sample	L *	a *	b *
T0	81.07	−1.94	6.70
T1	51.35	17.40	30.63
T2	60.36	12.91	27.38
T3	52.00	17.30	30.18
T4	60.34	11.58	25.84

L * for the lightness and a * and b * for the green–red and blue–yellow color components, respectively.

**Table 2:** Retention times, UV, MS, and MS^2^ data of phenolic acids and flavonols in the onion aqueous extract.

Peak Number	Compound	*t*_R_ (min)	UV λ_max_ (nm)	[M + H]^+^ (*m*/*z*)	MS^2^ (*m*/*z*)	[M − H]^−^ (*m*/*z*)	MS^2^ (*m*/*z*)
1	Protocatechuic acid glucoside	11.1	220, 260, 294	-	-	315	153 (100), 109 (25)
2	Protocatechuic acid ^1^	13.0	220, 260, 294	-	-	153	109 (100)
3	Vanillic acid ^1^	23.5	218, 260, 294	-	-	-	-
4	Quercetin 7,4′-diglucoside ^2^	31.6	254, 268sh, 366	627	465 (100), 303 (89)	625	463 (100), 301 (27),
5	Quercetin 3,4′-diglucoside ^2^	34.7	254, 266, 350	627	465 (100), 303 (53)	625	463 (100), 301 (14),
6	Quercetin 3-glucoside ^2^	36.9	256, 270sh, 374	465	303 (100)	463	301 (100), 191 (60)
7	Ellagic acid ^1^	42.2	254, 306sh, 370	-	-	301	-
8	Quercetin 4′-glucoside ^2^	42.8	254, 268sh, 366	465	303 (100)	463	301 (100)
9	Isorhamnetin 4′-glucoside ^2^	45.3	254, 374	479	317 (100)	477	315 (100), 300 (20)
10	Quercetin ^1^	48.9	256, 268sh, 370	303	285 (27), 257 (100), 229 (76), 165 (66), 201 (44), 187 (43)	301	151 (100), 179 (49), 187 (24), 164 (12), 239 (10)
11	Isorhamnetin ^1^	52.7	254, 374	317	302 (100), 285 (45), 257 (25), 139 (23), 135 (11)	315	300 (100)

Experimental conditions as described in Material and Methods. ^1^ Confirmed with reference standard. ^2^ The glycosylation site was tentatively assigned on the basis of literature data.

**Table 3:** Summary of the measured Folin–Ciocalteu, FRAP, and ORAC values of the onion extract and textiles.

**Sample**	**Type of Assay ^1^**		
	**FOLIN–CIOCALTEU**	**FRAP**	**ORAC ^2^**
	mg Eq GA/g onion skin	mg Eq TROLOX/g onion skin	
**EXTRACT**	78.50 ± 2.49	84.24 ± 6.10	181.66 ± 25.81
	mg Eq GA/g textile	mg Eq TROLOX/g textile	
**UW**	2.40 ± 1.82	0.05 ± 0.01	1.68
**T1**	21.56 ± 1.94	7.65 ± 1.43	13.60
**T2**	9.23 ± 0.61	2.20 ± 0.66	2.26
**T3**	21.20 ± 1.73	8.34 ± 1.86	13.84
**T4**	11.28 ± 0.49	3.28 ± 0.32	2.63

^1^ Data are expressed as mean ± SD. Analyses were performed in triplicate for each sample. ^2^ ORAC analysis on textiles was performed only once.

**Table 4:** LPO values determined using strip irradiated at 1 × 10^5^ µW/cm^2^ for 45 min. % LPO inhibition mean and standard deviation are reported. The meaning of P_0_, P_x_, A_0_, and A_x_ is explained in Section 3.11.

Volunteer	Application Period (h)	Po ^1^ (µM)	Px ^1^ (µM)	Ao ^1^ (µM)	Ax ^1^ (µM)	%LPO Inhibition
**A**	12	0.0333 ± 0.0295	0.6078 ± 0.0436	0.0293 ± 0.0213	0.1188 ± 0.1038	84.42
**B**		0.0276 ± 0.0270	0.5176 ± 0.0009	0.0070 ± 0.0030	0.0591 ± 0.0522	89.37
**A**	24	0.2512 ± 0.0351	0.3648 ± 0.0953	0.0480 ± 0.0388	0.0679 ± 0.0073	82.48
**B**		0.1287 ± 0.0389	0.3935 ± 0.0628	0.0506 ± 0.0330	0.1157 ± 0.0367	75.42

^1^ Data are expressed as mean ± SD.
